# Early Acceptability of a Mobile App for Contact Tracing During the COVID-19 Pandemic in France: National Web-Based Survey

**DOI:** 10.2196/27768

**Published:** 2021-07-19

**Authors:** Rajae Touzani, Emilien Schultz, Seth M Holmes, Stéphanie Vandentorren, Pierre Arwidson, Francis Guillemin, Dominique Rey, Alexandra Rouquette, Anne-Déborah Bouhnik, Julien Mancini

**Affiliations:** 1 Aix Marseille Univ, INSERM, IRD, SESSTIM, Sciences Economiques & Sociales de la Santé & Traitement de l’Information Médicale, ISSPAM, Equipe CANBIOS Labellisée Ligue Contre le Cancer Marseille France; 2 Institut Paoli-Calmettes, SESSTIM U1252 Marseille France; 3 CEPED, Université de Paris, IRD Paris France; 4 IMERA Mediterranean Institute for Advanced Study Marseille France; 5 Society and Environment Rausser College of Natural Resources University of California, Berkeley Berkeley, CA United States; 6 Medical Anthropology Department of Anthropology University of California, Berkeley Berkeley, CA United States; 7 School of Public Health University of California, Berkeley Berkeley, CA United States; 8 Santé Publique France Saint-Maurice France; 9 CIC 1433 Clinical Epidemiology, Inserm, CHRU, University of Lorraine Nancy France; 10 Public Health and Epidemiology Department, AP-HP, Bicêtre Hôpitaux Universitaires Paris Sud Le Kremlin-Bicêtre France; 11 Université Paris-Saclay, Univ. Paris-Sud, UVSQ, CESP, INSERM U1018 Villejuif France; 12 APHM, Hop Timone, BioSTIC, Biostatistique et Technologies de l’Information et de la Communication Marseille France

**Keywords:** COVID-19, mobile app, contact tracing, HLS19, health care disparities, public health

## Abstract

**Background:**

Several countries have implemented mobile apps in an attempt to trace close contacts of patients with COVID-19 and, in turn, reduce the spread of SARS-CoV-2. However, the effectiveness of this approach depends on the adherence of a large segment of the population.

**Objective:**

The aims of this study were to evaluate the acceptability of a COVID-19 contact tracing mobile app among the French population and to investigate the barriers to its use.

**Methods:**

The Health Literacy Survey 2019 questioned 1003 people in France during the COVID-19 pandemic on the basis of quota sampling. The survey collected sociodemographic characteristics and health literacy data, as well as information on participants’ communication with caregivers, trust in institutions, and COVID-19 knowledge and preventive behaviors. The acceptability of a mobile app for contact tracing was measured by a single question, the responses to which were grouped into three modalities: app-supporting, app-willing, and app-reluctant. Multinomial logistic regression analysis was performed to identify the factors associated with the acceptability of a mobile app during the COVID-19 pandemic.

**Results:**

Only 19.2% (193/1003) of all participants were app-supporting, whereas half of them (504/1003, 50.3%) were reluctant. The factors associated with willingness or support toward the contact tracing app included lower financial deprivation (app-willing: adjusted odds ratio [aOR] 0.8, 95% CI 0.69-0.93; app-supporting: aOR 0.7, 95% CI 0.58-0.84) and higher perceived usefulness of using a mobile app to send completed health questionnaires to doctors (app-willing: aOR 2.3, 95% CI 1.70-3.26; app-supporting: aOR 3.1, 95% CI 2.04-4.82). Furthermore, the likelihood of supporting the mobile app increased with age over 60 years (aOR 1.9, 95% CI 1.13-3.22), trust in political representatives (aOR 2.7, 95% CI 1.72-4.23), feeling concerned about the pandemic situation (aOR 2.2, 95% CI 1.47-3.32), and knowledge about the transmission of COVID-19 (aOR 2.0, 95% CI 1.39-2.96).

**Conclusions:**

The most socioeconomically precarious people, who are at a higher risk of SARS-CoV-2 infection, are also the most reluctant to using a contact tracing mobile app. Therefore, optimal adherence can only be effective with a targeted discourse on public health benefits to adopt such an app, which should be combined with a reduction in inequalities by acting on structural determinants.

## Introduction

Monitoring contacts of patients with COVID-19 is a key issue for long-term control of the pandemic. Several digital tools and eHealth applications have been deployed to effectively support health care systems in these efforts [1]. In particular, contact tracing apps have been designed to identify and inform people of likely exposure [2]. Since March, such contact tracing apps were installed by approximately 9.3% of the population around the world [3] and adopted by many countries, including China, South Korea, Australia, Turkey, Germany, Israel, and Singapore [3], with mandatory or voluntary setups. In France, the governmental decision—following the advice of its scientific board—was to develop a dedicated app called “StopCovid”; this app has been available for download on mobile phones since early June 2020 [4]. In October 2020, the StopCovid app was updated and renamed “TousAntiCovid” [5]. When app users indicate that they have been infected with COVID-19, the app uses Bluetooth technology to recover information of all close contacts (ie, all other TousAntiCovid users who have spent more than 15 minutes within a distance of 1 meter of the said user, as recorded over a period of 14 days) and alerts them with generic notifications, recommending them to quarantine themselves and to take a COVID-19 screening test. There have been numerous criticisms focused on data privacy [6,7] and technical limitations of the app software [8]. Because downloading and using the TousAntiCovid app is voluntary in France, its effectiveness is dependent on acceptability and adoption among users.

Beyond the technical considerations, such contact tracing apps require high adherence rates among the population to be effective. According to a previous study, at least 56% of a population must use a digital contact tracing app in order to control the pandemic [9]. However, another study reported that app-based tracing was more efficient than conventional tracing even with 20% coverage [2]. It is therefore necessary to convince a maximum number of citizens of their interest in using such an app and to remove potential barriers to app use. However, the factors that determine the acceptance of contact tracing apps remain largely unknown. Beyond the lack of information as a barrier, social deprivation that increases risk and severity of COVID-19 might also relate to potential barriers to the use of a contact tracing app [10]. For this reason, it is also important that the introduction of the apps does not inadvertently create or exacerbate social inequalities [11].

The objective of this study is to determine the acceptability of a contact tracing mobile app, as well as the potential barriers to its use.

## Methods

The data analyzed here has been sourced from the French Health Literacy Survey 2019 (HLS19), which was set up as part of France’s participation in the World Health Organization Action Network project Measuring Population and Organizational Health Literacy (M-POHL) [12]. HLS19 was administered to a sample of the adult French population 2 weeks after the end of the first lockdown in France (ie, between May 27 and June 5, 2020) at the time of the official launch of the TousAntiCovid app (June 2, 2020). After this complete lockdown, several other national or regional lockdown periods were announced in France in 2020-2021. The sample of 1000 internet users aged 18-75 years was drawn from an access panel, respecting the French population structure for sex, age, regions, and area of residence (urban or rural). After informed consent was obtained, the web-based survey collected information on respondents’ sociodemographic characteristics (gender, age, level of education, and region of residence), perception of health, health literacy, and navigation in the health system. In addition, the survey collected additional national items such as communication with caregivers through new technologies, perception of medical research, trust in institutions, as well as data about the COVID-19 pandemic. The study methodology was reviewed and approved by the Ethics Evaluation Committee of the French National Health and Medical Research Institute (CEEI, IRB 00003888).

In this study, we focused on analyzing the sociodemographic data of the survey respondents. A financial deprivation score was calculated by combining 3 questions related to financial abilities: (1) to pay all bills at the end of the month, (2) to buy drugs if needed, and (3) to pay for medical examinations and treatments that are not covered by health insurance (response scale: very easy, easy, difficult, and very difficult). The mean score was transformed by multiplying it by (5/3) [13]. The scores ranged from 0 to 5, with higher scores indicating higher financial deprivation [13].

Health literacy level was calculated using the 16-item version of the European Health Literacy Survey Questionnaire (HLS-EU-Q16). Responses to the survey items were recorded using a 4-point Likert-type scale [14]. For the score, the modalities were dichotomized into easy and difficult categories, by merging “very easy” and “easy” responses, as well as “difficult” and “very difficult” responses. This allowed us to have 3 groups according to the health literacy score: inadequate (HLS-EU score: <9), problematic (HLS-EU score: 9-12), and adequate (HLS-EU score: >12), with a maximum possible score of 16 [14].

The level of knowledge on the transmission of COVID-19 (KT-COVID-19) was measured by combining the correct answers to the following two questions: (1) “In your opinion, can someone who shows no sign of the disease transmit the coronavirus?” and (2) “In your opinion, are protective behaviors effective in limiting the spread of the coronavirus?” A perfect level of KT-COVID-19 involved answering “Yes, definitely” to both questions.

Trust in institutions was measured using three questions asking about the participants’ level of trust in doctors, scientists, and political representatives. For each question, the responses were grouped into two modalities: yes (very and rather trustworthy) and no (rather not and not at all trustworthy).

The participants’ acceptability of a mobile app to follow close contact between people during an epidemic was measured by the following question: “In your opinion, is it acceptable to use mobile phones to study close contact between people during an epidemic?” with 5 possible modalities: “Yes, definitely,” “Yes, probably,” “No, probably not,” “No, definitely not,” and “I don’t know.” We grouped the last three modalities as *app-reluctant* and named the other two modalities *app-willing* (“Yes, probably”) and *app-supporting* (“Yes, definitely”).

Chi-square tests and analysis of variance (ANOVA) were used for descriptive analyses, depending on the type of variables. A multinomial logistic model was used to identify the factors associated with the acceptability of a mobile app to study close contact between people during an epidemic. After adjustment for age, a stepwise procedure was performed to select significant factors in the model (entry threshold; *P*<.20). The significance threshold in multivariate analyses was set at 5%. All analyses were performed using the STATA software program (version 14.0; StataCorp LLC).

## Results

### Description of the Study Sample

A description of our study sample is presented in Table 1. Overall, 1003 French adults responded to the survey. Half of the participants were women (n=506, 50.4%), and half (n=510, 50.9%) were 46 years old or younger. A little more than half of all participants (n=592, 59.0%) had a high level of education. A small proportion of participants reported they had been infected with COVID-19 themselves (n=50, 5.0%) or that someone in their household had been infected (n=58, 5.8%). In terms of health literacy, 39.8% (n=399) of the participants had an HLS-EU-Q16 score reflecting problematic (n=264, 26.3%) or inadequate (n=135, 13.5%) levels (ie, HLS-EU score: ≤12). Of note, most respondents (n=664, 66.2%) had an imperfect level of KT-COVID-19. With regard to our variable of interest, 50.3% (n=504) of all participants were app-reluctant (“No, probably not acceptable”: n=183, 18.3%; “No, definitely not acceptable”: n=216, 21.5%; and “Don’t know”: n=105, 10.5%) and only 19.2% (n=193) were app-supporting (“Yes, definitely acceptable”).

**Table 1:** Description of the characteristics of the study sample (N=1003).

Characteristic		Participants
**Age (years), n (%)**		
	18-35	291 (29.0)
	36-46	219 (21.9)
	47-59	253 (25.2)
	60-75	240 (23.9)
**Gender, n (%)**		
	Women	506 (50.4)
	Men	497 (49.6)
**Place of birth, n (%)**		
	France	951 (94.8)
	Other country	52 (5.2)
**Education level, n (%)**		
	Less than high-school degree	172 (17.2)
	High-school degree	239 (23.8)
	Higher education	592 (59.0)
**Area of residence, n (%)**		
	Rural	212 (21.1)
	Urban	791 (78.9)
Financial deprivation score (range: 0-5)^a^, mean (SD)		1.7 (1.1)
**Health literacy level (HLS-EU-Q16^b^ score range: 0-16), n (%)**		
	Inadequate (<9)	135 (13.5)
	Problematic (9-12)	264 (26.3)
	Adequate (>12)	604 (60.2)
**Infected with COVID-19, n (%)**		
	Yes	50 (5.0)
	No	862 (85.9)
	I don’t know	91 (9.1)
**A household member infected with COVID-19, n (%)**		
	Yes	58 (5.8)
	No	851 (84.8)
	I don’t know	94 (9.4)
**Significantly concerned about the situation caused by COVID-19, n (%)**		
	Yes	266 (26.5)
	No	737 (73.5)
**KT-COVID-19^c^ level, n (%)**		
	Perfect	339 (33.8)
	Imperfect	664 (66.2)
**Acceptability of a mobile app to study close contact between people during an epidemic^d^, n (%)**		
	Yes, definitely	193 (19.2)
	Yes, probably	306 (30.5)
	No, probably not	183 (18.3)
	No, definitely not	216 (21.5)
	Don’t know	105 (10.5)

^a^A financial deprivation score was calculated by combining answers to 3 questions. Higher scores indicate higher financial deprivation.

^b^HLS-EU-Q16: European Health Literacy Survey Questionnaire, 16-item.

^c^KT-COVID-19: knowledge on the transmission of COVID-19 (measured by combining answers to 2 questions on the transmission of COVID-19 and on the effectiveness of barrier gestures; a perfect level of KT-COVID-19 corresponds to a correct answer to both questions).

^d^This variable has been grouped into 3 modalities: app-supporting (“Yes, definitely”), app-willing (“Yes, probably”), and app-reluctant (“No, probably not”; “No, definitely not”; and “Don’t know”).

### Factors Associated With the Acceptability of Contact Tracing Using Mobile Phones

Results of the univariate analysis (Table 2) showed no significant association between app-reluctance and sociodemographic factors except for financial deprivation, with participants reluctant to use such apps reporting higher financial deprivation scores (*P*<.001).

App-supporting participants (75/193, 38.9%) felt significantly more concerned by the situation caused by COVID-19 than app-willing participants (80/306, 26.1%), who in turn felt more concerned than app-reluctant participants (111/504, 22.0%; *P*<.001). The positive gradient was also observed between the acceptability of contact tracing apps and avoiding close contact in the past week with people they did not live with (app-reluctant 334/504, 66.3%; app-willing: 229/306, 74.8%; app-supporting: 147/193, 76.2%; *P*=.006). The level of KT-COVID-19 was also significantly associated with participants’ attitudes toward such apps; for instance, 53.4% (103/193) of app-supporting participants had a perfect KT-COVID-19 level compared to 29.4% (148/504) of app-reluctant participants (*P*<.001).

Moreover, trust in political representatives (*P*<.001), scientists (*P*=.02), and doctors (*P*=.006) was positively associated with the acceptability of a contact tracing app during a pandemic. We also observed a positive association concerning the perceived usefulness of digital technologies: during medical consultations (broadcasting and recording), to complete and send health assessment questionnaires, or to make a medical appointment (all *P*<.001).

**Figure 1:**
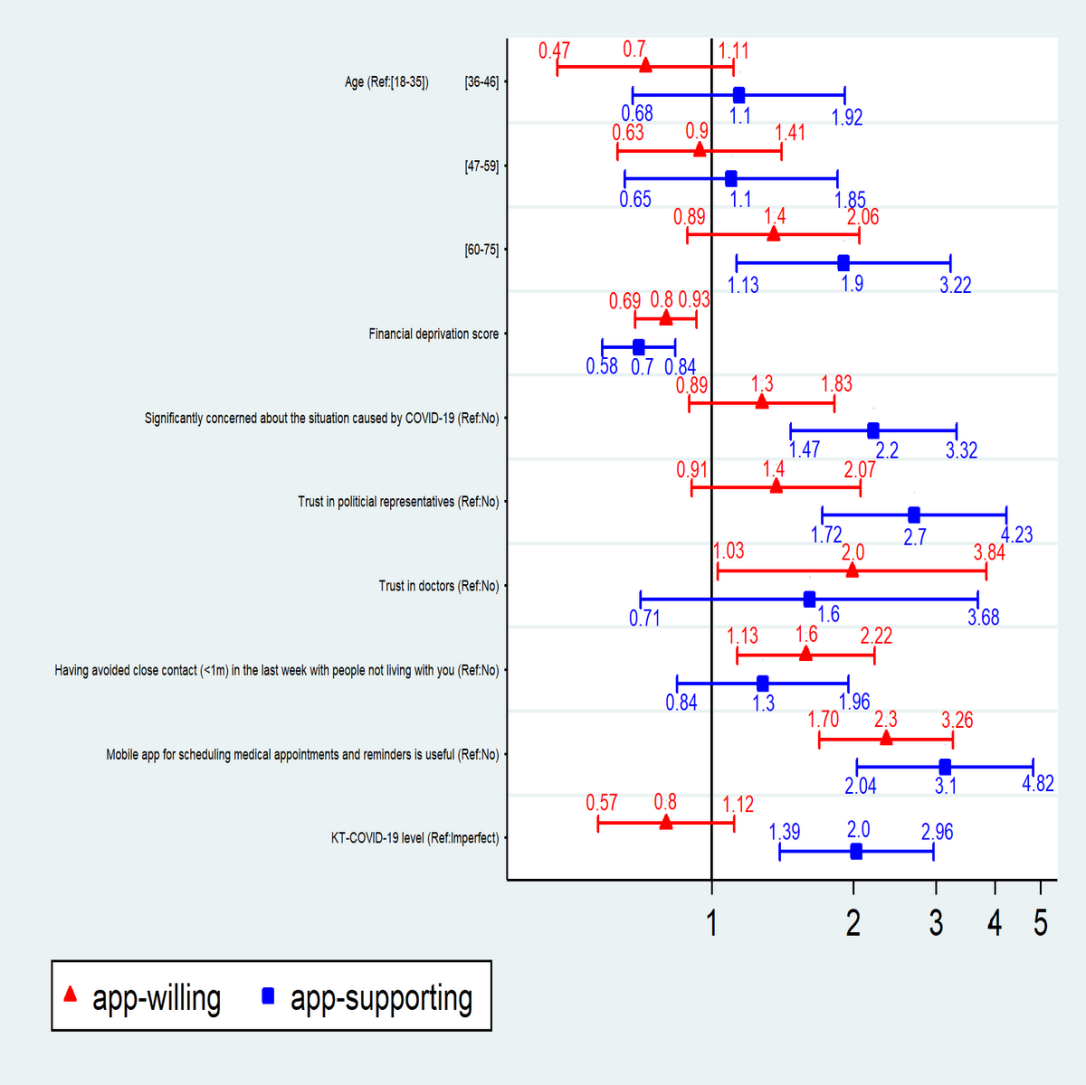
Factors associated with acceptability of a mobile app to study close contact between people during an epidemic (N=1003). Dots and whiskers represent adjusted odds ratio and 95% CI levels from multivariate analyses for definite (“app-supporting,” blue) or probable (“app-willing,” red) acceptability of a contact tracing app. KT-COVID-19: knowledge on the transmission of COVID-19; Ref: referent category in the model.

After adjusting for age (Figure 1), the two groups not reluctant to use a contact tracing app were found to be associated with a lower level of financial deprivation and with higher perceived usefulness of a mobile app to send doctors answers to health questionnaires. The likelihood of a participant’s willingness to use a contact tracing app increased among those who trusted doctors and those who had avoided close contact with other people in the past week. App-supporters were 60 years and older, felt more concerned about the situation of the COVID-19 pandemic, trusted political representatives, and had a perfect level of KT-COVID-19.

**Table 2:** Factors associated with the acceptability of a mobile app to study close contact between people during an epidemic: univariate analysis (N=1003).

Factor			App-reluctant (n=504)		App-willing (n=306)		App-supporting (n=193)		*P* value^a^	
**Age (years), n (%)**										.11
	18-35	146 (29.0)		95 (31.1)		50 (25.9)				
	36-46	120 (23.8)		54 (17.7)		45 (23.3)				
	47-59	134 (26.6)		76 (24.8)		43 (22.3)				
	60-75	104 (20.6)		81 (26.5)		55 (28.5)				
**Gender, n (%)**										.35
	Women	265 (52.6)		145 (47.4)		96 (49.7)				
	Men	239 (47.4)		161 (52.6)		97 (50.3)				
**Place of birth, n (%)**										.16
	France	476 (94.4)		287 (93.8)		188 (97.4)				
	Other country	28 (5.6)		19 (6.2)		5 (2.6)				
**Education level, n (%)**										.73
	Less than high-school degree	90 (17.9)		55 (18.0)		27 (14.0)				
	High-school degree	122 (24.2)		72 (23.5)		45 (23.3)				
	Higher education	292 (57.9)		179 (58.5)		121 (62.7)				
**Area of residence, n (%)**										.26
	Rural	117 (23.2)		57 (18.6)		38 (19.7)				
	Urban	387 (76.8)		249 (81.4)		155 (80.3)				
Financial deprivation score (range: 0-5)^b^, mean (SD)			1.9 (1.1)		1.6 (0.9)		1.4 (1.1)		*<.001*	
**Health literacy level (HLS-EU-Q16^c^ score range: 0-16), n (%)**										*.046*
	Inadequate (<9)	76 (15.1)		38 (12.4)		21 (10.9)				
	Problematic (9-12)	140 (27.8)		65 (21.2)		59 (30.6)				
	Adequate (>12)	288 (57.1)		203 (66.3)		113 (58.5)				
**Infected with COVID-19, n (%)**										.83
	Yes	22 (4.4)		18 (5.9)		10 (5.2)				
	No	438 (86.9)		261 (85.3)		163 (84.5)				
	I don’t know	44 (8.7)		27 (8.8)		20 (10.4)				
**A household member infected with COVID-19, n (%)**										.49
	Yes	23 (4.6)		23 (7.5)		12 (6.2)				
	No	431 (85.5)		257 (84.0)		163 (84.5)				
	I don’t know	50 (9.9)		26 (8.5)		18 (9.3)				
**Significantly concerned about the situation caused by COVID-19, n (%)**										*<.001*
	Yes	111 (22.0)		80 (26.1)		75 (38.9)				
	No	393 (78.0)		226 (73.9)		118 (61.1)				
**Trust in political representatives, n (%)**										*<.001*
	Yes	62 (12.3)		56 (18.3)		61 (31.6)				
	No	442 (87.7)		250 (81.7)		132 (68.4)				
**Trust in scientists, n (%)**										*.02*
	Yes	438 (86.9)		278 (90.9)		181 (93.8)				
	No	66 (13.1)		28 (9.1)		12 (6.2)				
**Trust in doctors, n (%)**										*.006*
	Yes	457 (90.7)		293 (95.8)		185 (95.9)				
	No	47 (9.3)		13 (4.2)		8 (4.1)				
**Having avoided close contact (<1 m) in the last week with people not living with you, n (%)**										*.006*
	Yes	334 (66.3)		229 (74.8)		147 (76.2)				
	No	170 (33.7)		77 (25.2)		46 (23.8)				
**Broadcasting of the consultation by video so that relatives who are not present can participate is useful, n (%)**										*<.001*
	Yes	162 (32.2)		136 (44.4)		95 (49.2)				
	No	342 (67.9)		170 (55.6)		98 (50.8)				
**Video recording of consultations to remember its content is useful, n (%)**										*<.001*
	Yes	201 (39.9)		164 (53.6)		118 (61.1)				
	No	303 (60.1)		142 (46.4)		75 (38.9)				
**Mobile app for scheduling medical appointments and reminders is useful, n (%)**										*<.001*
	Yes	393 (78.0)		269 (87.9)		170 (88.1)				
	No	111 (22.0)		37 (12.1)		23 (11.9)				
**Mobile app for sending your doctor answers to questionnaires assessing your health is useful, n (%)**										*<.001*
	Yes	284 (56.3)		225 (73.5)		153 (79.3)				
	No	220 (43.7)		81 (26.5)		40 (20.7)				
**KT-COVID-19^d^ level, n (%)**										*<.001*
	Perfect	148 (29.4)		88 (28.8)		103 (53.4)				
	Imperfect	356 (70.6)		218 (71.2)		90 (46.6)				

^a^Italicized values indicate statistical significance.

^b^A financial deprivation score calculated by combining answers to 3 questions. Higher scores indicate higher financial deprivation.

^c^HLS-EU-Q16: European Health Literacy Survey Questionnaire, 16-item.

^d^KT-COVID-19: knowledge on the transmission of COVID-19 (measured by combining answers to 2 questions on the transmission of COVID-19 and on the effectiveness of barrier gestures; a perfect level of KT-COVID-19 corresponds to a correct answer to both questions).

## Discussion

### Principal Findings

Even amidst vaccine rollout and with a significant risk of multiple epidemic waves or a possible shift toward a long-term pandemic, contact tracing is an important public health strategy to control the spread of the COVID-19 pandemic [15], and it can be effective if it is adopted by a large segment of the population. In our study, the proportion of people who would agree to use such a mobile app for tracing and follow-up of close contacts in the context of COVID-19 was found to be 49.7% (499/1003), including 306 participants responding “Yes, probably” and 193, “Yes, definitely”.

Similar figures were noted before the implementation of TousAntiCovid app in a sample of the French population aged over 15 years (N=1051), wherein 49% of the study population indicated that they intended to install the app, of which only 15% were certain and 34% probably claimed to do so [16]. An acceptability rate of 38.4% was also reported in a recent study (May 07, 2020, N=1849) [17]. Despite relatively high theoretical acceptability, after the launch of the mobile app, only 3.1% of the people in France downloaded it (as of mid-July 2020) [3], as confirmed by 73.5% (737/1003) of our study participants reporting that they did not feel very concerned about the situation caused by COVID-19. Additional recent data further confirm the low adoption of TousAntiCovid [18], downloaded by approximately 20% of the French population in March 2021 [19]. Moreover, we do not know if this population indeed used the app after downloading it, as no data are available regarding use and uninstallation. Recent results from the literature confirm this difference between the actual and theoretical rate of app use in the case of voluntary installation in various countries, including Australia (70% agreed to use the app vs 44% downloaded it) [20], United States (55% accepted vs 21% downloaded it) [21], and Germany (81% accepted vs 36% downloaded it) [22]. In countries where the installation of the app was mandatory, we observed a very high download rate (eg, 91% in Qatar) [23]. Several reasons might explain this large gap between theoretical acceptability and actual use. For instance, technical and financial considerations might limit access to the app. Therefore, special efforts are needed to help people who are more financially precarious; it may also be important to better inform people about the availability of such a tool and its usefulness in controlling the pandemic. Indeed, apart from the content and availability of the messages, many factors can be associated with app reluctance, such as the hierarchy of needs (food or administrative insecurity), discrimination and fear of losing one's job, worries about being isolated, and losing social support. To improve the acceptability of such a tool, special attention must be paid to protect people against the risks and difficulties they might be facing.

Several factors were identified to play a role in the potential acceptability of a contact tracing app. Our results show that people aged 60-75 years would support such an app during the COVID-19 pandemic, contrary to what has been demonstrated in the literature with regard to technology acceptance, where advanced age is associated with a lower level of acceptability of mobile apps [24]. However, this finding is consistent with early evidence on the acceptability of contact tracing apps reported by a German study [25]. Indeed, in the context of the COVID-19 pandemic, the population at higher risk is typically over 60 years old [26], and people who perceive themselves at risk might be more inclined to adopt such an app. Our results confirm that agreeing to use the contact tracing app requires people to be concerned about the situation created by the pandemic and/or to avoid close contacts outside their household. At the start of the pandemic, the communication by the French government and the scientific community sometimes included contradictory messages that led to confusion (eg, whether or not to wear a mask, COVID-19 is not an alarming virus) [27]. Because contact tracing has been framed as a long-term solution, it could have also been seen as less essential than treatments or wearing masks. Contradictory communication likely played a role in the disinterest on the part of the population and the lack of collective awareness. Indeed, knowledge about COVID-19 transmissibility through asymptomatic individuals and about the effectiveness of barrier gestures was low in our sample (339/1003, 33.8%).

Another aspect to consider here is people’s trust in institutions. Contact app tracing is intrinsically linked to the state response to the pandemic. As such, attitudes toward institutions should influence the acceptability of an app. Our analysis shows that trusting politicians and doctors has a positive effect on people’s intention to use the contact tracing mobile app. Those who trust political representatives’ express absolute certainty, unlike those who trust doctors who are almost certain. A study conducted on the general population during the COVID-19 lockdown showed that one of the reasons for nonadherence with the app was the concern of the use of this technology for the purposes of government oversight [28]. In this study, we do not know if this is the reason people mistrust political representatives, but we assume that the issue of personal data privacy plays a role in the negative relationship between trust in the government and likelihood of installing the app [29], particularly in a context where individual rights are more generally restricted. Moreover, the conflicting messages from the government (including the fact that the Prime Minister and two other ministers of the French government had not downloaded the TousAntiCovid app [30]) and the medical controversies about drug treatment during the COVID-19 pandemic have further weakened the population’s trust in institutions. These factors seem to have served to enrich conspiracy theories related to COVID-19. Marinthe et al [31] demonstrated that people with a conspiracy mentality are less willing to comply with government-driven preventive behaviors during the COVID-19 outbreak. These results are in line with those obtained in a French study [17], unlike an American study where trust in politicians was not correlated with app use [21]. These authors explained this difference by the fact that at the time of their study, various American political parties supported the use of the app.

Lack of familiarity with technologies is also an obstacle to using a contact tracing app. Our results show that app-reluctant individuals are those who consider that electronic patient-reported outcomes (ePRO) are not useful. One study indicated that patients with cancer who did not use ePROs were those who expressed lower acceptability for mobile technology and, therefore, lower adherence to mobile health [32]. The positive link between attitude toward technologies and its acceptance has previously been demonstrated in the context of the COVID-19 pandemic [33]. There is a need to better understand the link between the public understanding of science and technology, their overall acceptability, and the consequences for health practices, especially in an emergency context where uncertainty prevails.

Our results also show that the likelihood of agreeing to use mobile tracking technology during the COVID-19 pandemic increases with financial resources and health literacy. At the same time, the people most vulnerable to COVID-19 are also those whose precariousness is the most marked by their socioeconomic situation (income, professional activity, and origin) with frequent inadequate levels of health literacy [34]. We then assume that people with lower health literacy might have different risk perceptions and are probably not reached by adequate and understandable preventive recommendations. Several studies have shown that people with low health literacy have more difficulty finding information and understanding COVID-19–related messages [35,36]. Our results are in line with these previous findings. Indeed, our findings show clearly that adoption of a behavior of social utility—in this case, using a contact tracing app—was associated with a perfect level of knowledge of the modes of transmission of COVID-19. Studies have also shown that digital solutions are often less used by people with low levels of health literacy or those who do not have access to the internet [37,38]. More socioeconomically precarious people often have poor internet access and may face difficult living conditions (housing conditions, employment, access to running water, etc) that could affect their ability to follow the recommended guidelines. Hence, unequal access to information and its understanding, as well as the level of risk perception, could play a major role in citizens’ adherence to new COVID-19 prevention technologies. It is therefore clear that COVID-19 highlights pre-existing health inequalities and can even accentuate them through the deployment of the contact tracing app. This is why specific interventions are needed to fight against these inequalities. Targeting this group of the population with understandable messages on the usefulness of the app and addressing the specific reasons for their reluctance might help increase its use, but it is clearly not enough. More should be done to decrease inequities overall and specifically in relation to the implementation of such apps. Broader approaches that intervene not only on individual and interpersonal factors but also upon structural determinants are needed [39]. In particular, structural interventions should at longer term seek to change the global context that fosters social and health inequalities. If such contact tracing apps are needed to fight against pandemics, every willing person should be able to access such technology and to use it without potential negative consequences.

### Limitations and Strengths

Our study has some limitations. The use of the close contact tracing app was studied through theoretical acceptability. This type of study upstream of the implementation of a new technology can help adapt the actions but should be followed, after the implementation of the app, by additional studies on real acceptability and usage. Moreover, we do not know whether participants have a smartphone with adequate data allowance and battery performance to use the app; despite these reasons, our web-based survey was conducted among a highly educated sample (59% in this study compared to 37% in the French population [40]) of internet users, which is not representative of the entire French population because there are still numerous households that do not have internet access.

Finally, one of the strengths of this study is the rather large size of the sample and the national representativeness with regard to age group, gender, area of residence, and population density, which was achieved by quota sampling. This large sample size gave us enough statistical power to detect the detrimental effect of financial deprivation and imperfect health literacy despite surveying a socioeconomically privileged sample.

### Conclusions

To our knowledge, this is the first study in France to evaluate the impact of financial deprivation on the acceptability of a close contact tracing mobile app used during the COVID-19 outbreak on a representative quota sample. Knowing the characteristics of the people who do not adhere to the new tracking technology in the context of a pandemic is essential to adopting effective strategies. Combining the tracing tool with testing and isolation can significantly facilitate the fight against the virus. Strong adherence to this technology would not be possible if public authorities do not conduct extensive public awareness campaigns to foster trust in institutions and to clarify what the app does, and importantly, what it does not do, particularly among more precarious people. Globally, the current COVID-19 health crisis reinforces the need to fight against social inequities generally and specifically to provide to all people not only masks or vaccines but also technologies that are useful to control the spread of transmissible diseases.

## Abbreviations

ANOVA: analysis of variance
ePRO: electronic patient-reported outcomes
HLS19: Health Literacy Survey 2019
HLS-EU-Q16: European Health Literacy Survey Questionnaire, 16-item
KT-COVID-19: knowledge on the transmission of COVID-19
M-POHL: Measuring Population and Organizational Health Literacy

## References

[1] Fagherazzi G, Goetzinger C, Rashid MA, Aguayo GA, Huiart L (2020). Digital health strategies to fight COVID-19 worldwide: challenges, recommendations, and a call for papers. J Med Internet Res.

[2] Kretzschmar ME, Rozhnova G, Bootsma MCJ, van Boven M, van de Wijgert JHHM, Bonten MJM (2020). Impact of delays on effectiveness of contact tracing strategies for COVID-19: a modelling study. The Lancet Public Health.

[3] Chan S (2020). COVID-19 contact tracing apps reach 9% adoption in most populous countries. SensorTower.

[4] (2020). TousAnitCovid app. Webpage in French.

[5] (2020). “TousAntiCovid”, new version of the StopCovid app: what's changing? Webpage in French. Live Well Digital.

[6] (2020). Bahrain, Kuwait and Norway contact tracing apps among most dangerous for privacy. Amnesty.

[7] Saw YE, Tan EY, Liu JS, Liu JC (2021). Predicting public uptake of digital contact tracing during the COVID-19 pandemic: results from a nationwide survey in Singapore. J Med Internet Res.

[8] Horton O (2020). French app StopCovid still facing hurdles amid EU concerns about data access. Radio France Internationale (RFI).

[9] Hinch R, Probert W, Nurtay A, Kendall M, Wymant C, Hall M, Lythgoe K, Cruz A, Zhao L, Stewart A, Ferretti L, Parker M, Meroueh A, Mathias B, Stevenson S, Montero D, Warren J, Mather N, Finkelstein A, Bonsall D, Fraser C (2020). Effective configurations of a digital contact tracing app: a report to NHSX.

[10] Williamson EJ, Walker AJ, Bhaskaran K, Bacon S, Bates C, Morton CE, Curtis HJ, Mehrkar A, Evans D, Inglesby P, Cockburn J, McDonald HI, MacKenna B, Tomlinson L, Douglas IJ, Rentsch CT, Mathur R, Wong AYS, Grieve R, Harrison D, Forbes H, Schultze A, Croker R, Parry J, Hester F, Harper S, Perera R, Evans SJW, Smeeth L, Goldacre B (2020). Factors associated with COVID-19-related death using OpenSAFELY. Nature.

[11] Thorneloe R, Epton T, Fynn W, Daly M, Stanulewicz N, Kassianos A, Shorter G, Moll S, Campbell M, Sodergren S, Chapman S, Sutherland L, Armitage C, Arden M, Chater A, Byrne-Davis L, Hart J Scoping review of mobile phone app uptake and engagement to inform digital contact tracing tools for COVID-19. PsyArXiv..

[12] M-POHL - Action Network on Measuring Population and Organizational Health Literacy of EHII - WHO-Europe.

[13] Dietscher C, Pelikan J (2019). The action network for measuring population and organizational health literacy (M-POHL) and its Health Literacy Survey 2019 (HLS19). Eur J Public Health.

[14] Rouquette A, Nadot T, Labitrie P, Van den Broucke S, Mancini J, Rigal L, Ringa V (2018). Validity and measurement invariance across sex, age, and education level of the French short versions of the European Health Literacy Survey Questionnaire. PLoS One.

[15] Atlani-Duault L, Chauvin F, Yazdanpanah Y, Lina B, Benamouzig D, Bouadma L, Druais PL, Hoang A, Grard M, Malvy D, Delfraissy J (2020). France's COVID-19 response: balancing conflicting public health traditions. The Lancet.

[16] Martelli-Banégas D, Desreumaux M, Favré T (2020). Questions spécifiques : perceptions de l’application STOPCOVID et regards sur l’enjeu du partage des données personnelles. Rapport de résultats – Mai 2020. Report in French. Harris Interactive.

[17] Guillon M, Kergall P (2020). Attitudes and opinions on quarantine and support for a contact-tracing application in France during the COVID-19 outbreak. Public Health.

[18] Lévèque L (2020). Checknews - How many times has the StopCovid app been downloaded? How much does it cost? For what results? Webpage in French. Libération.

[19] Grably R (2021). Since its launch, the TousAntiCovid application has alerted 100,000 contact cases. Webpage in French. BFM Business.

[20] Garrett PM, White JP, Lewandowsky S, Kashima Y, Perfors A, Little DR, Geard N, Mitchell L, Tomko M, Dennis S (2021). The acceptability and uptake of smartphone tracking for COVID-19 in Australia. PLoS One.

[21] Hargittai E, Redmiles EM, Vitak J, Zimmer M (2020). Americans’ willingness to adopt a COVID-19 tracking app. First Monday.

[22] Blom AG, Wenz A, Cornesse C, Rettig T, Fikel M, Friedel S, Möhring K, Naumann E, Reifenscheid M, Krieger U (2021). Barriers to the large-scale adoption of a COVID-19 contact tracing app in Germany: survey study. J Med Internet Res.

[23] Jacob S, Lawarée J (2020). The adoption of contact tracing applications of COVID-19 by European governments. Policy Design and Practice.

[24] Rai A, Chen L, Pye J, Baird A (2013). Understanding determinants of consumer mobile health usage intentions, assimilation, and channel preferences. J Med Internet Res.

[25] Grill E, Eitze S, DeBock F, Dragano N, Huebl L, Schmich P, Wieler L, Betsch C Sociodemographic characteristics determine download and use of a corona contact tracing app in Germany - results of the COSMO surveys. PsychArchives..

[26] (2020). COVID-19: epidemiological update of August 27, 2020. Santé Publique France. Webpage in French.

[27] Schultz E, Ward JK, Atlani-Duault L, Holmes SM, Mancini J (2021). French public familiarity and attitudes toward clinical research during the COVID-19 pandemic. Int J Environ Res Public Health.

[28] Milsom L, Abeler J, Altmann S, Toussaert S, Zillessen H, Blasone R (2020). Survey of acceptability of app-based contact tracing in the UK, US, France, Germany and Italy. OSF.

[29] (2020). Why is the StopCovid app debated? Webpage in French. Le Journal du Net (JDN).

[30] Lausson J (2020). Jean Castex estime qu'il n'a pas besoin d'utiliser l'application StopCovid. Webpage in French. Numerama.

[31] Marinthe G, Brown G, Delouvée S, Jolley D (2020). Looking out for myself: Exploring the relationship between conspiracy mentality, perceived personal risk, and COVID-19 prevention measures. Br J Health Psychol.

[32] Blake H (2008). Mobile phone technology in chronic disease management. Nurs Stand.

[33] Jansen S, Hurmuz M, Den Ouden M, van Velsen L Predictors to use mobile apps for monitoring COVID-19 symptoms and contact tracing: a survey among Dutch citizens. medRxiv..

[34] Dubost C-L, Pollak C, Rey S (2020). Social inequalities in the face of the Covid-19 epidemic - State of play and perspectives. Webpage in French. DREES (Directorate of Research, Studies, Evaluation and Statistics).

[35] McCaffery K, Dodd R, Cvejic E, Ayrek J, Batcup C, Isautier J, Copp T, Bonner C, Pickles K, Nickel B, Dakin T, Cornell S, Wolf M (2020). Health literacy and disparities in COVID-19-related knowledge, attitudes, beliefs and behaviours in Australia. Public Health Res Pract.

[36] Wolf MS, Serper M, Opsasnick L, O'Conor RM, Curtis LM, Benavente JY, Wismer G, Batio S, Eifler M, Zheng P, Russell A, Arvanitis M, Ladner D, Kwasny M, Persell SD, Rowe T, Linder JA, Bailey SC (2020). Awareness, attitudes, and actions related to COVID-19 among adults with chronic conditions at the onset of the U.S. outbreak: a cross-sectional survey. Ann Intern Med.

[37] Nguyen A, Mosadeghi S, Almario CV (2017). Persistent digital divide in access to and use of the internet as a resource for health information: results from a California population-based study. Int J Med Inform.

[38] Triana AJ, Gusdorf RE, Shah KP, Horst SN (2020). Technology literacy as a barrier to telehealth during COVID-19. Telemed J E Health.

[39] Brown AF, Ma GX, Miranda J, Eng E, Castille D, Brockie T, Jones P, Airhihenbuwa CO, Farhat T, Zhu L, Trinh-Shevrin C (2019). Structural interventions to reduce and eliminate health disparities. Am J Public Health.

[40] Teachers - Students per teaching staff. OECD (Organisation for Economic Co-operation and Development) Data.

